# Alagille Syndrome: A Novel Mutation in *JAG1* Gene

**DOI:** 10.3389/fped.2019.00199

**Published:** 2019-05-15

**Authors:** Rita Fischetto, Viviana V. Palmieri, Maria E. Tripaldi, Alberto Gaeta, Angela Michelucci, Maurizio Delvecchio, Ruggiero Francavilla, Paola Giordano

**Affiliations:** ^1^Clinical Genetics Unit, Department of Paediatric Medicine, Giovanni XXIII Children's Hospital, Bari, Italy; ^2^Pediatric Section, Department of Biomedicine and Human Oncology, University A. Moro, Bari, Italy; ^3^PediatricRadiology Unit, Giovanni XXIII Children's Hospital, Bari, Italy; ^4^Laboratory of Molecular Genetics, University Hospital of Pisa, Pisa, Italy; ^5^Pediatrics Unit, “Madonna delle Grazie” Hospital, Matera, Italy

**Keywords:** Alagille syndrome, *JAG1*, stop codon mutation, Next Generation Sequencing, hypertransaminasemia

## Abstract

Alagille syndrome is an autosomal dominant multisystem disorder with variable phenotypic penetrance, caused by heterozygous mutations in *JAG1* or *NOTCH2*, encoding for the components of the Notch signaling pathway. In this paper, we described a novel mutation not yet reported in literature. This 3-years old male child was referred to our Clinical Genetics Unit because of delayed psychomotor development, systolic murmur, dysmorphic facial features, and hypertransaminasemia. The novel *JAG1* heterozygous *c.2026delT* variant in exon 16 was found. *JAG1* mutations are classified as protein truncating and non-protein truncating, without any genotype-phenotype correlation. The detected mutation determines a stop codon (p.Cys676AlafsTer67) in the gene sequence, encoding a truncated protein. Our report broadens the spectrum of *JAG1* gene mutations.

## Background

Alagille syndrome (ALGS; OMIM 118450) is an autosomal dominant multisystem disorder with variable phenotypic penetrance first described in 1969 by Daniel Alagille (1). The incidence rate is 1:70,000–100,000 live births (2, 3). Molecular diagnosis has increased the number of cases detected and the true incidence is probably close to 1 in 30,000 (1, 4). Although clinical features may differ significantly, the diagnosis of ALGS is mainly based on clinical findings (5, 6). Initial diagnosis is based on the presence of intrahepatic bile duct paucity and at least of 3 other clinical features: chronic cholestasis, cardiac disease (pulmonary stenosis), ocular abnormalities (posterior embryotoxon), skeletal abnormalities (butterfly-like vertebrae), and peculiar facial features (broad forehead, deep-set eyes, bulbous nose, and small pointed mandible) (7–9). Patients have a high prevalence of renal and vascular disease as well (1).

ALGS is caused by mutations in genes that impair Notch signaling, a highly conserved pathway that is fundamental to the transcription of genes for cell fate and differentiation of multiple organ systems (10–14). Almost all cases of ALGS are caused by mutations in the *JAG1* gene (20p12.2), which consists of 26 exons, while a small rate of patients has a heterozygous mutation in the Notch receptor*, NOTCH2* gene (1p13) (15–18). Although mutations causing ALGS have now been identified, diagnostic challenges remain because there are not genotype–phenotype correlations (19). Albeit several genotype-phenotype correlation studies have been performed, they have not shown a link between mutation type and clinical manifestation or severity, leading to the hypothesis that a second gene could work to modify the effects of a *JAG1* or *NOTCH2* mutation (1). The diagnosis is based on clinical features. Liver biopsy typically shows paucity of the intrahepatic bile ducts, but it is no longer considered mandatory to make a diagnosis of ALGS, and the presence of cholestasis is acceptable to fulfill this criterion. Confirmatory diagnosis is based on gene sequencing. However, about 4% of patients with clinical diagnosis of ALGS may not show any genetic variant, suggesting that further genetic mechanisms are still to be elucidated (1).

In this paper, we describe a patient carrying a novel mutation not yet reported in literature. Signed informed consent has been acquired from the patient's parents for the publication of this case report and any potentially identifying information was removed.

## Case Report

The patient is a Caucasian 3-years old male child, late-preterm born (36 weeks) from vaginal delivery, after a pregnancy complicated by placental detachment. Birth weight was 2,490 g (26° centile). He was the first child of an unrelated couple. Family history was negative for cardiac or hepatic disorders. The main stages of psychomotor development were delayed (sitting position at 8 months with hypotonia; walking at 18 months; speaking at 3 years). At 20 months of age a systolic murmur was found at the cardiac auscultation and heart ultrasound was performed, showing a mild stenosis of the pulmonary branches. Screening for metabolic diseases was negative, except for the finding of hypertransaminasemia. Because of dysmorphic facial features, delayed neurological development and elevated liver enzymes, a genetic condition was suspected and the patient was referred to the Clinical Genetics Unit of the Giovanni XXIII Children's Hospital in Bari.

**Figure 1:**
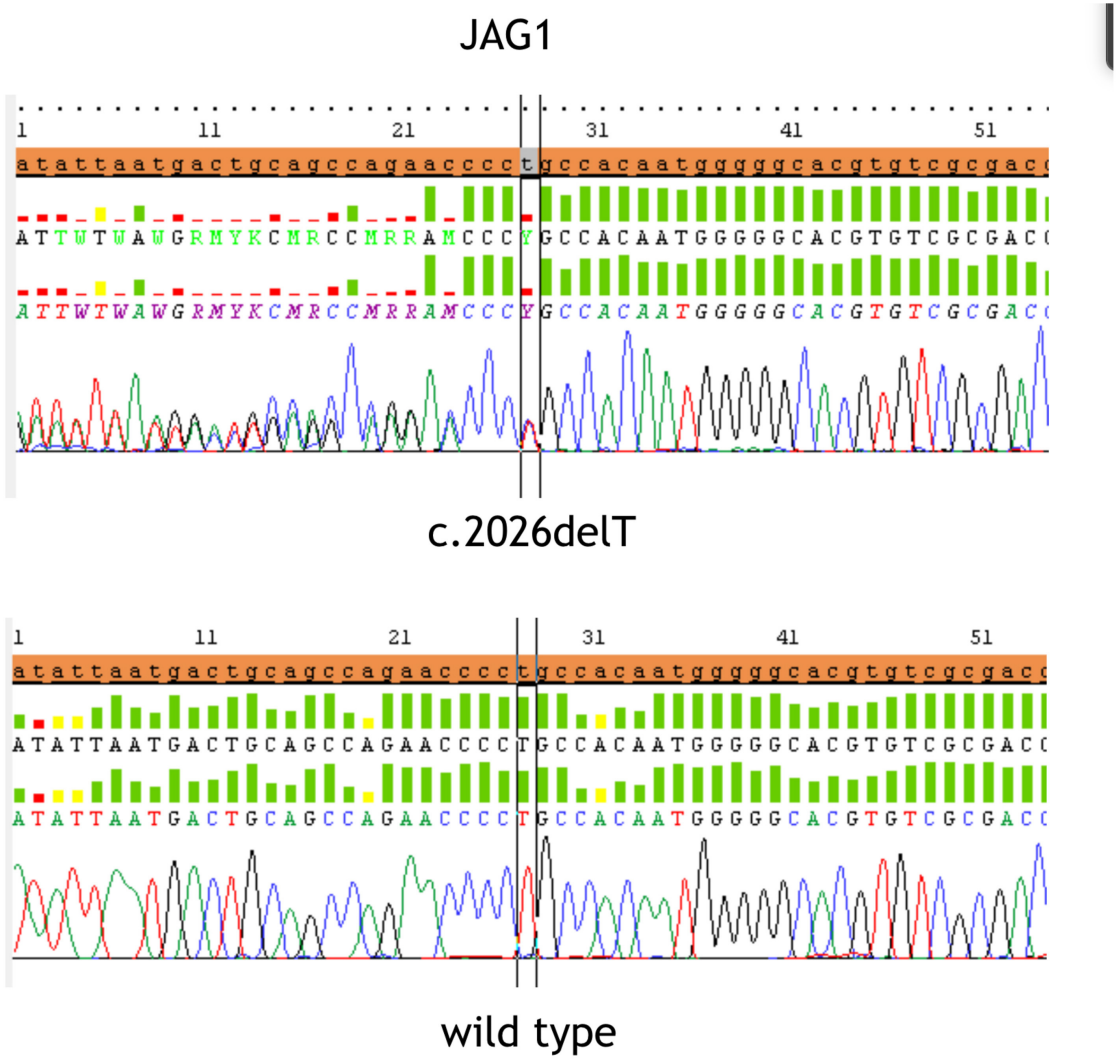
The figure displays the heterozygous sequence variant c.2026delT (p.Cys676AlafsTer67) in exon 16 identified and confirmed by Sanger sequencing in the upper part and the wild type sequence at the bottom.

At referral, height, weight and head circumference were normal (>50° centile). He featured prominent frontal bossing, saddle nose with a bulbous tip, 2/VI systolic cardiac murmur, severe psychomotor retardation suggesting an autistic phenotype. His stools were hypocholic with remains of undigested food. Fundus oculi and brain resonance were normal. Karyotype and FRAXA analysis resulted negative. After patient's parents signed the informed consent, gene sequencing of *JAG1* (NM_000214) was performed by Next Generation Sequencing. Target enrichment was done by TruSeq custom amplicon (Illumina, San Diego, CA, United States) according to the manufacturer's instructions. Template library was prepared and was sequenced using MiseqIllumina platform (Illumina, San Diego, CA, United States). Annotation and filtering of variants were performed with Illumina Variant Studio version 2.0, following recommended settings. To evaluate the completeness of the method for the screening of the targeted gene, the sequencing coverage of each amplicon was analyzed in detail using Integrative Genome Viewer version 2.3 (Broad Institute, Cambridge, MA). Variants and region with a depth coverage below 30x were confirmed by Sanger sequencing. The heterozygous sequence variant c.2026delT; p.Cys676AlafsTer67 in exon 16 was identified and confirmed using Sanger sequencing (Figure 1), also because the coverage in exon 16 was very low (<30 x). Primers sequences for PCR amplification of the exon 16 were designed using Primer3 software: Forward primer CCTGTCGTGAATGGTCCTG, Reverse primer CCAGGCCCAGAGAAATATCA. The variant was absent in both parents, arisen as a *de novo* variant, which determines the formation of a stop codon. Variant was checked for previously reported causative mutations in published works and mutation databases: Human Gene Mutation Database (HGMD) and Leiden Open Variation Database (LOVD) and it has never been described before. Moreover, variant was searched indbSNP, 1,000 Genomes and ExACdatbases, to exclude common single nucleotide polymorphism.

The child started symptomatic therapy with ursodeoxycholic acid and multidisciplinary follow-up, in particular rehabilitative psychomotor follow-up.

## Discussion

Alagille syndrome is a highly variable, autosomal dominant disorder, which involves multiple organ systems. Table 1 shows a summary of the clinical characteristics and its frequency in patients with ALGS. Its management requires a multidisciplinary team, including mainly specialists in medical genetics, gastroenterology, hepatology, nutrition, cardiology, ophthalmology, and many others (20, 21). The main clinical and pathological features are chronic cholestasis, characteristic facial features, cardiac defect, minor abnormalities of vertebral segmentation, ophthalmologic abnormalities, and dysplastic kidneys (20). The liver disease typically causes severe debilitating pruritus and disfiguring xanthomas, which require treatment with ursodeoxycholic acid and other medications such as cholestyramine, rifampin, and naltrexone (22). Infants and children with ALGS are often diagnosed based on their clinical features: chronic cholestasis is the most common presenting attribute of patients, but dysmorphic facial features are considered one of the best ways to diagnose ALGS (5). The differential diagnosis included Progressive Familiar Cholestasis (PFIC), childhood primary sclerosing cholangitis, congenital hepatic fibrosis, childhood autoimmune hepatitis, childhood primary biliary cirrhosis, alpha-1 antitripsin deficiency, cystic fibrosis and other diseases in which paucity of bile ducts occurs (3).

**Table 1:** A summary of the clinical features and the frequency reported among individuals with ALGS.

**Common system involved**	**Overall frequency**	**Frequency of Finding in *JAG1*+ ALGS**	**This case**
Liver: Paucity of biliary duct, conjugated hyperbilirubinemia and liver failure	Up to 100%	100%	√
Heart: Structural changes, pulmonary stenosis, and tetralogy of Fallot	90–97%; 60–67%, and 7–16%	100%	√
Facial features: Prominent forehead, deep-set eyes with moderate hypertelorism, pointed chin, and saddle or straight nose with a bulbous tip	20–97%	97%	√
Eye: Posterior embryotoxon	78–89%	75%	X
Bones: Vertebral anomalies (butterfly vertebra)	33–93%	64%	Not available
Kidney: Ureteropelvic obstruction and renal tubular acidosis	39%	40%	X

*Adapted from Saleh et al. (20). √: present; X: not present*.

ALGS is caused by heterozygous mutations in 1 of 2 genes that are fundamental components of the Notch signaling pathway, JAGGED1 and Notch 2. Mutations in *JAG1* gene were first identified more than 20 years ago, and above 500 mutations have been reported (1, 13, 16, 23, 24). The spectrum of *JAG1* mutations includes more frequently protein-truncating mutations (75%), and non-protein truncating mutations (25%) (Tables 2A,B) (1, 24). Sequencing all exons and the immediately adjacent intronic regions to identify splice site mutations allows to identify the majority of *JAG1* and *NOTCH2* mutations. Because mutations in *JAG1* are predominant, sequencing of this gene occurs first followed by deletion or duplication analysis via multiplex ligation-dependent probe amplification, chromosomal microarray, or fluorescence *in situ* hybridization. Sequencing of *JAG1* identifies approximately 85% of ALGS mutations, and deletion/duplication analysis yields an additional approximately 9% of molecular diagnoses. In the absence of an identified mutation in *JAG1*, sequencing of *NOTCH2* identifies another 2 to 3% of mutations in ALGS. Up to date large deletion or duplication mutations in *NOTCH2* have not been reported. A causative mutation for the remaining 2 to 4% of clinically diagnosed ALGS patients has not been identified yet, and the application of various next-generation sequencing techniques could help identify a molecular origin in this population (1). Unfortunately no genotype-phenotype correlation exists between clinical manifestations and the specific *JAG1* pathogenic variant or the location of the mutation within gene (19). Although genetics of ALGS is well-defined, there is a very variable expressivity of the disease. Individuals with the same mutations, including patients belonging to the same family, show discordance in the phenotype (1). In support of this concept, the genotype-phenotype correlation studies did not identify a link between the mutation type and clinical manifestation or severity (18). In view of the absence of an identified environmental factor that influences the severity of the disease or presentation, scientists hypothesized that a second gene could work modifying the effects of a *JAG1* or *NOTCH2* mutation to worsen or improve the disease features. Several studies have identified putative genetic modifiers to help explain the variable expressivity of this disease (1).

**Table 2A:** Type and frequency of *JAG1* mutations found in ALGS.

	**Mutation type**	**Reported (n)**	**Frequency (%)**
Protein truncating	Small deletions	150	28.4
	Small duplications/insertions	102	19.4
	Non sense	85	16.1
	Gross deletions	47	8.9
	Indels	12	2.3
Non-protein truncating	Missense	81	15.4
	Splice site	47	8.9
	Gross duplications/insertions	2	0.4
	Translocations	1	0.2
Total		527	100

*Adapted from Mitchell et al. (1)*.

**Table 2B:** Exons more frequently involved per genetic mutation (Results for variants in the *JAG1* gene reported in the ClinVar database).

**Exons more frequentlyinvolved per genetic mutation**
Deletions: Exon 2,7,16,17,23,25
Duplications: Exon 3,8,11,13,17,18,23,24
Insertions: Exon 8, 23
Stop codon mutations: Exon 3,4,6,20,21,23,24
Missense: Exon 1,2,4,5,9,20
Splicing mutations: Exon 16

The patient described in this paper was referred to our Unit because of psycho-motor delay, autistic pattern, facial dysmorphic features, and hypertransaminasemia. Thus, at referral beside the typical liver involvement which is the main feature of ALGS, the clinical features of our patient were facial features, heart disease, and severe neurological involvement; the less frequent clinical features in ALGS were not present in our case (Table 1).

The *JAG1* gene sequencing showed the novel*c.2026delT* variant in exon 16, which determines a stop codon (p.Cys676AlafsTer67) in the gene sequence. As a result, this mutation produces a truncated protein. Although exon 16 has already been described as a site of disease-causing mutations, this variant has not been reported yet in literature. Further studies could clarify the effect of this mutation, but the stop codon causes the premature truncation of the amino acids sequence. In the same position, it was previously reported the *de novo* c.2026T>G variant leading to the p.C676G missense mutation which was disease-causing according to the Mutation Tester and probably damaging according to the PolyPhen-2 (25). The mutation was found in a male with 4 clinical features (cholestasis, cardiac murmur, skeletal abnormalities, characteristic face). The majority of point and frameshift mutations could be detected by sequencing 11 exons (exons 3, 5, 6, 11, 14, 16, 18, 21, and 23–25). It has been reported that exon 16 is involved mostly by deletions and splicing mutations, due to a single nucleotide substitution (such c.2071T>A, c.2078G>A, and c.2091G>A) or small duplications (c.2070_2073dupCTGT) (25, 26). In this paper we describe a patient with Alagille syndrome carrying a frameshift mutation in the exon 16 of *JAG1* gene. This exon is frequently involved by disease-causing mutations in Alagille syndrome.

## Concluding Remarks

Our report broadens the spectrum of mutations in the *JAG1* gene. Broadening the mutations spectrum may help genetists to better identify etiological mutations leading to ALGS and overall it could provide further insights to identify the hotspots exons where mutations happen more frequently.

## Ethics Statement

Signed informed consent has been acquired from the patient's parents for the publication of this case report and any potentially identifying information was removed. Gene sequencing was performed after informed consent was signed by the patient's parents.

## Author Contributions

RiF and RuF were in charge of clinical follow-up of the patient. VP and MT drafted the paper. AG is the radiologist who performed the imaging. AM is the biologist who ran the gene sequencing. MD and PG reviewed the paper and gave substantial contribution to data interpretation.

### Conflict of Interest Statement

The authors declare that the research was conducted in the absence of any commercial or financial relationships that could be construed as a potential conflict of interest.
